# A label-free biosensor based on graphene and reduced graphene oxide dual-layer for electrochemical determination of beta-amyloid biomarkers

**DOI:** 10.1007/s00604-020-04267-x

**Published:** 2020-04-25

**Authors:** Jagriti Sethi, Michiel Van Bulck, Ahmed Suhail, Mina Safarzadeh, Ana Perez-Castillo, Genhua Pan

**Affiliations:** 1 Wolfson Nanomagnetics Laboratory, School of Engineering, Computing and Mathematics, University of Plymouth, Devon, PL4 8AA UK; 2 Instituto de Investigaciones Biomédicas (CSIC-UAM), Arturo Duperier, 4, 28029 Madrid, Spain; 3 Centro de Investigación Biomédica en Red sobre Enfermedades Neurodegenerativas (CIBERNED), Valderrebollo, 5, 28031 Madrid, Spain

**Keywords:** Graphene, Aβ_1–42_ detection, Alzheimer’s disease, Electrochemical biosensors, Screen-printed electrodes

## Abstract

A label-free biosensor is developed for the determination of plasma-based Aβ_1–42_ biomarker in Alzheimer’s disease (AD). The platform is based on highly conductive dual-layer of graphene and electrochemically reduced graphene oxide (rGO). The modification of dual-layer with 1-pyrenebutyric acid *N*-hydroxysuccinimide ester (Pyr-NHS) is achieved to facilitate immobilization of H31L21 antibody. The effect of these modifications were studied with morphological, spectral and electrochemical techniques. The response of the biosensor was evaluated using differential pulse voltammetry (DPV). The data was acquired at a working potential of ~ 180 mV and a scan rate of 50 mV s^−1^. A low limit of detection (LOD) of 2.398 pM is achieved over a wide linear range from 11 pM to 55 nM. The biosensor exhibits excellent specificity over Aβ_1–40_ and ApoE ε4 interfering species. Thus, it provides a viable tool for electrochemical determination of Aβ_1–42_. Spiked human and mice plasmas were used for the successful validation of the sensing platform in bio-fluidic samples. The results obtained from mice plasma analysis concurred with the immunohistochemistry (IHC) and magnetic resonance imaging (MRI) data obtained from brain analysis.

**Electronic supplementary material:**

The online version of this article (10.1007/s00604-020-04267-x) contains supplementary material, which is available to authorized users.

**Graphical abstract:**
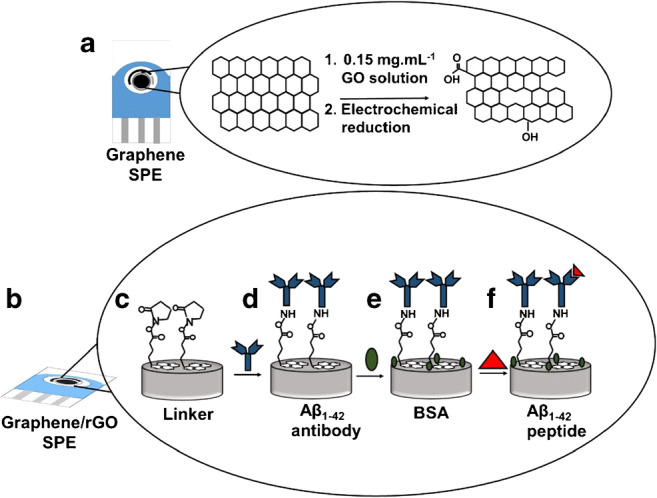
Schematic representation of the electrochemical system proposed for Aβ_1–42_ determination: (a) modification of graphene screen-printed electrode (SPE) with monolayer graphene oxide (GO) followed by its electrochemical reduction generating graphene/reduced graphene oxide (rGO) dual-layer (b), modification of dual-layer with linker (c), Aβ_1–42_ antibody (H31L21) (d), bovine serum albumin (BSA) (e) and Aβ_1–42_ peptide (f).

## Introduction

The current trend in the realm of medical science is to investigate the molecular basis of ailments as opposed to following a symptomatic approach. This has prompted remarkable improvements in research for chronic ailments such as Alzheimer’s disease (AD). Abnormal levels of certain biomarkers in bio-fluids have been associated with AD progression [1]. One such biomarker is beta-amyloid (Aβ) peptide consisting of 40 and 42 amino acids, namely Aβ_1–40_ and Aβ_1–42_. Their abnormal levels in bio-fluids have been correlated with amyloid pathology in AD-affected brain [2]. The continuous monitoring of Aβ levels could provide aid in early diagnosis, before the onset of symptoms [3]. As a result, several platforms including enzyme-linked immunosorbent assay (ELISA) [4], surface-enhanced spectroscopy (SERS) [5], surface plasmon resonance (SPR) [6], electrochemical sensors [7–9] and field effect transistors [10] have been developed. Among these, electrochemical sensors are most attractive and, therefore, widely preferred. This is attributed to various merits such as high sensitivity [3], possibility of label-free sensing, portability [11], simplicity of apparatus and easy handling [9]. However, electrochemical platforms developed for Aβ_1–42_ detection have shown various limitations. These include lack of bio-fluid analysis, low sensitivity and non-specific bindings particularly with Aβ_1–40_ [12].

An interdigitated microelectrode system was recently developed for the detection of Aβ_1–42_. The electrode was used alongside a signal cancellation and amplification processing system (SCAP) [3]. The platform was validated with mice plasma samples. Despite excellent sensitivity, use of an additional SCAP system makes the platform more complex and expensive. The specificity of the sensor is also questionable towards Aβ_1–42_ in the presence of interfering Aβ_1–40_ and ApoE species [13]. Another biosensor based on gold (Au) electrode and mercaptopropionic acid self-assembled monolayer (SAM) reported high sensitivity [2]. However, the platform failed to depict specificity against any interfering species present in the complex bio-fluids. With Aβ_1–42_/Aβ_1–40_ ratios gaining interest as a potential biomarker, reliable detection of individual biomarker is more important than ever [14, 15]. In addition, deposition of Aβ_1–42_ starts earlier compared with Aβ_1–40_ during AD progression [16]. The interference with ApoE ε4 species can also prompt false results when analysing Aβ_1–42_ in patients’ samples. This is due to the fact that ApoE ε4 can be present in up to 10,000-fold excess in human plasma [17]. Therefore, specific determination of Aβ biomarkers is an important prerequisite for bio-fluid analysis and understanding AD progression.

Graphene is a widely investigated material due to its remarkable properties such as high conductivity, biocompatibility, ease of surface functionalization [18], large surface to volume ratio and low environmental impact [19]. Graphene-based biosensors can detect extremely low concentration of biomarkers [1]. This is attributed to the fact that presence or absence of a very few analyte molecules can trigger a recognizable change in electrical properties of graphene. Another material which is widely preferred for biosensors is reduced graphene oxide (rGO). It has structural similarities to graphene with large number of electroactive sites providing highly sensitive material for sensing [18]. Most of the graphene-based biosensors are based on either only graphene [20] or only rGO [21]. A combination of the two materials for the detection of protein biomarkers is yet to be explored. The conductivity of rGO on graphene is much higher than on other materials such as carbon [22]. Coupling of rGO and graphene provides high conductivity with large number of available active sites which can be useful for biosensing. The rGO surface can be chemically functionalized to anchor antibodies on the surface [23] which can be achieved by covalent or non-covalent modifications. The covalent modification may damage and degrade the properties of rGO [24]. Therefore, non-covalent modification methods are a preferred choice. 1-Pyrenebutyric acid *N*-hydroxysuccinimide ester (Pyr- NHS) is a bi-functional molecule which binds non-covalently to graphene and carbon surfaces [25, 26]. It promotes strong immobilization of antibodies without any adverse effect on the underlying structure. This has shown to improve sensitivity for detection of clusterin, another well-known biomarker of AD [25]. Thus, Pyr-NHS is an excellent linker for the immobilization of probes on the graphene biosensors.

In the present work, graphene/rGO dual-layer screen-printed electrode (SPE) is developed for rapid, label-free and reliable detection of Aβ_1–42_. Higher redox current is observed for dual-layer SPE, confirming its improved sensitivity compared with only graphene and graphene/GO SPE. The immobilization of H31L21 antibody achieved via Pyr-NHS leads to high specificity of the biosensor towards Aβ_1–42_ peptides without damaging underlying rGO structure. The interaction of Pyr-NHS with rGO on a dual-layer surface is reported for the first time. The platform shows wide linear dynamic range with low detection limit. To the best of our knowledge, this is the first report of a biosensor depicting specificity against Aβ_1–40_ and ApoE ε4 interfering species as well as high sensitivity. The biosensor has been successfully validated with both spiked human and mice plasma samples. Detailed comparison of dual-layer SPE over the existing label-free biosensors are made in Table 1.

**Table 1:** An overview of recently reported label-free electrochemical methods for determination of Aβ_1–42_

Substrate	Materials used	Detection technique	LOD	Specificity studies	Matrices	References
Au electrode	Au NPs/mercaptopropionic acid SAM	SWV	1.15 pM	–	PBS	[2]
SiO_2_	Glutaraldehyde/PVP-CHO/3-aminopropyl triethoxysilane	Impedance analysis, noise cancellation system	–	Prostate-specific antigen and brain-derived neurotrophic factor	PBS, Plasma	[3]
Au ICE	EDC-NHS/6-mercaptohexanoic acid SAM	EIS	15.5 pM, 20 pM^*^	Tumour necrosis factor-alpha, insulin, C-reactive protein	PBS, serum	[8]
Carbon printed chip	Protein G/16-mercaptohexadecanoic acid SAM/Au NPs	EIS	570 pM	–	PBS	[11]
GCE	AuNPs/MWCNTs	DPV	28 pM	–	Rat brain (in vivo)	[27]
Au film	EDC-NHS/3-mercaptohexanoic acid SAM	DPV	–	Tau	PBS, serum	[28]
Graphene/rGO SPE	Pyr-NHS	DPV	2.398 pM	Aβ_1–40_ and ApoE ε4	PBS, Plasma	This work

^*^Values were converted from pg mL^−1^ to pM

## Experimental

### Reagents and animals

Single-layer graphene oxide dispersion in water was purchased from Graphene Supermarket (USA) (https://graphene-supermarket.com/). Chemicals such as bovine serum albumin (BSA), 1-pyrenebutyric acid *N*-hydroxysuccinimide ester (Pyr-NHS), phosphate buffered saline (PBS), human plasma, Aβ_1–42_ peptides, potassium ferricyanide (K_3_Fe(CN)_6_) and potassium chloride (KCl) at biochemical grade were purchased from Sigma-Aldrich (Dorset, UK) (https://www.sigmaaldrich.com/united-kingdom.html). Aβ_1–42_ antibody (H31L21), 4′,6-Diamidino-2-Phenylindole (DAPI) and Alexa Fluor 546 goat anti-rabbit secondary antibody were provided by Thermo Fisher Scientific (https://www.thermofisher.com/uk/en/home.html). Aβ_1–40_ and ApoE ε4 peptides were obtained from Tocris (UK) (https://www.tocris.com/). Paraformaldehyde (PFA) was purchased from Merck (Spain) (https://www.merckgroup.com/es-es). Sucrose, Triton X-100 and Eppendorf® LoBind Microcentrifuge Tubes were obtained from Sigma-Aldrich (Spain) (https://www.sigmaaldrich.com/spain.html). Normal Goat Serum (NGS) and Vectashield H-1200 were provided by Vector Laboratories (Spain) (https://vectorlabs.com/). Microvette® CB 300 K2E tubes were obtained from Sarstedt (Spain) (https://www.sarstedt.com/en/home/).

Blood and brain samples were obtained from 9 and 12 months old wild-type (WT) and transgenic (Tg) animals of the strain B6.Cg-Tg (APPswe/PSEN1dE9) 85Dbo/Mmjax, Lab Stock# 005864. All the experiments related to the animals were performed in the Centro de Investigación Biomédica en Red Enfermedades Neurodegenerativas (CIBERNED), Spain. The ethical approval was provided by the “Ethics Committee for Animal Experimentation” of the Instituto de Investigaciones Biomédicas (CSIC-UAM) and experiments were carried out in accordance with European Communities Council Directive (2010/63/EEC) and National regulations (Normative 53/2013).

Plasma studies with biosensors were performed in the University of Plymouth, UK, with approval of Local Ethics Committee.

### Apparatus

Graphene-modified SPEs and μStat potentiostat were provided by Metrohm DropSens (UK). The electrode had graphene as working, carbon as an auxiliary and silver as a reference electrode. Electrochemical cyclic voltammetry (CV) and differential pulse voltammetry (DPV) measurements were performed at room temperature with a μStat potentiostat. The experiments were controlled using DropView 8400 2.0 software. The μStat cable connector (ref. DRP-CAST) was used as an interface between the potentiostat and SPEs.

Raman spectra were obtained using XPLORA HORIBA system and Olympus BX41 microscope. The system used a 532-nm laser source, power of 100 mW, × 100 objective lens, a scan range of 1100 to 2000 cm^−1^ and an exposure time of 5–60 s.

Immunofluorescence data was collected using a Nikon Eclipse 90i microscope using Plan APO 4x objective, equipped with Nikon DS-Fi1 digital camera. It was connected to Nis-Elements software (Madrid, Spain).

Magnetic resonance imaging (MRI) was performed on a Bruker Pharmascan Biospect system (Bruker Medical Gmbh, Ettlingen, Germany). The system was equipped with a 7.0-T horizontal-bore superconducting magnet, ^1^H receive-only mouse brain surface coil and volume transmission coil and a Bruker gradient insert (maximum intensity 36 G cm^−1^). All data was acquired using a Hewlett-Packard console running on Paravision 5.1 software (Bruker Medical Gmbh, Germany). Sensor with monitor system (SA Instruments, Stony Brook, NY) was used to measure the rate and depth of respiration.

The Thermo Scientific™ Nexsa™ system was used for carrying out the X-ray photoelectron spectrum (XPS) analysis. The morphological analysis was done with JEOL 6610 LV scanning electron micrscope (SEM) from Oxford Instruments.

### Fabrication of biosensor

**Fig. 1:**
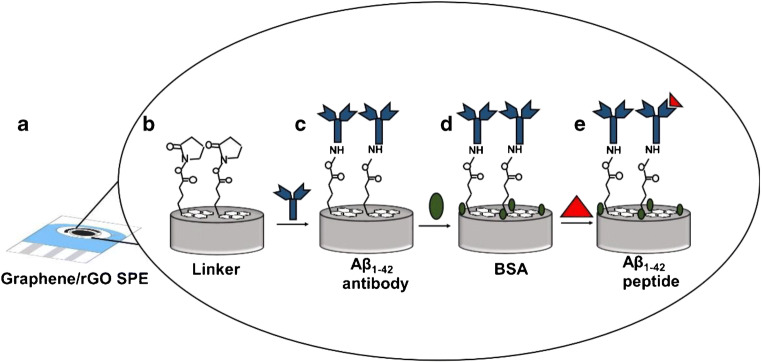
Schematic representation of the electrochemical system for detection of Aβ_1–42_: **a** graphene/rGO SPE-modified with linker (**b**), antibody (**c**), BSA (**d**) and Aβ_1–42_ peptide (**e**)

The graphene/rGO dual-layer SPE was prepared using the protocol mentioned in our previous work [18]. Briefly, an aqueous solution of 0.15 mg mL^−1^ GO was prepared in deionized (DI) water. The solution was then carefully drop casted onto the surface and dried for 2 h at room temperature. This promotes strong bond formation of GO on graphene SPE. After that, the SPE was washed 3 times with DI water. GO layer was then electrochemically reduced in 10 mM K_3_[Fe(CN)_6_] containing 1 M KCl solution by one CV cycle. A scan rate of 100 mV s^−1^ was applied, and potential was varied between 0.5 and -1.5 V [18]. In the next step, the dual-layer SPE was incubated in 5 mM Pyr-NHS (in methanol) for 2 h at room temperature. Subsequently, it was incubated overnight (16 h) in 20 μg mL^−1^ of Aβ_1–42_ antibody (H31L21) at 4 °C. Then, electrodes were rinsed with PBS to remove any unbound antibody. This was followed by blocking of the surface with 2% BSA (in PBS) for 2 h at room temperature. The schematic representation of the fabrication process is described in Fig. 1.

### Interaction of Aβ_1–42_ with the sensor

The desired dilutions of Aβ_1–42_ peptides (0.2 pM to 55 nM) were freshly prepared in PBS by sonication for 2 min. The prepared peptides were kept on ice during the experiments to avoid their aggregation. Then, 20 μL of solution was drop casted onto the modified SPE and incubated at room temperature for 60 min. After that, the sensor was washed 3 times before the measurements to remove any unbound peptide. The measurement time for one sensor is approximately 3–4 min.

### Blood sampling method

Blood was drawn by submandibular bleeding methods using a sterile blood lancet [29]. The blood droplets were collected by Microvette® CB 300 K2E tubes. A novel standardized method of blood extraction and purification protocol was developed to obtain non-haemolytic plasma samples from rodents. It consisted of two centrifuge steps. The first centrifuge step (1500 ×*g* for 10 min at 15 °C) was performed soon after the blood collection, to separate the plasma fraction from red blood cells and buffy coat. Then, the plasma fraction was collected in Eppendorf® LoBind Microcentrifuge Tubes and directly placed on ice. The second centrifuge step (3500 ×*g* for 10 min at 4 °C) was carried out to separate additional red blood cell debris. Finally, the plasma was divided in aliquots and stored at − 80 °C for long-term preservation.

### Plasma sample analysis

Dual-layer SPE was validated with both mice and human plasma. The plasma from human was spiked with desired concentration of Aβ_1–42_. Then, 20 μl of spiked plasma was drop casted on to the biosensors for 60 min. Mice plasma samples were analysed in a similar way without any pretreatment.

### Electrochemical detection

All the measurements were carried out in an electrolyte solution containing 10 mM potassium ferricyanide solution with 1 M KCl as the supporting electrolyte. CV was recorded from − 0.2 to + 0.5 V potential and 0.05 V s^−1^ scan rate without the application of any preconditioning potential or accumulation time. For DPV, potential was measured from − 0.15 to + 0.45 V with a step potential of 0.01 V, pulse amplitude of 0.05 V, pulse period of 0.4 s and a scan rate of 0.05 V s^−1^.

### Immunohistochemistry (IHC)

Double immunofluorescence analysis was performed with brain sections of animals, obtained as previously described [30]. Briefly, animals were anesthetized followed by transcardial perfusion. After this, brains were extracted and postfixed with 4% PFA and 30% sucrose solution overnight at 4 °C. Subsequently, 30 μm coronal section were obtained using a cryostat. The selected free floating cortical-hippocampal section was blocked in 0.1 M phosphate buffer (PB) containing 3% NGS and 0.1% Triton X-100 for 1 h at room temperature. It was then incubated with Aβ_1–42_ primary antibody (H31L21), overnight at 4 °C. Several washes were performed in 0.1 M PB containing 0.1% Triton X-100. Then, the sections were incubated for 1 h with a secondary antibody, Alexa Fluor 546 goat anti-rabbit. Nuclei staining was performed using DAPI. Finally, the brain sections were mounted with Vectashield H-1200, and fluorescence microscope images were performed. The images were processed by ImageJ software using stitching method [31].

## Results and discussion

### Characterization of the biosensor

**Fig. 2:**
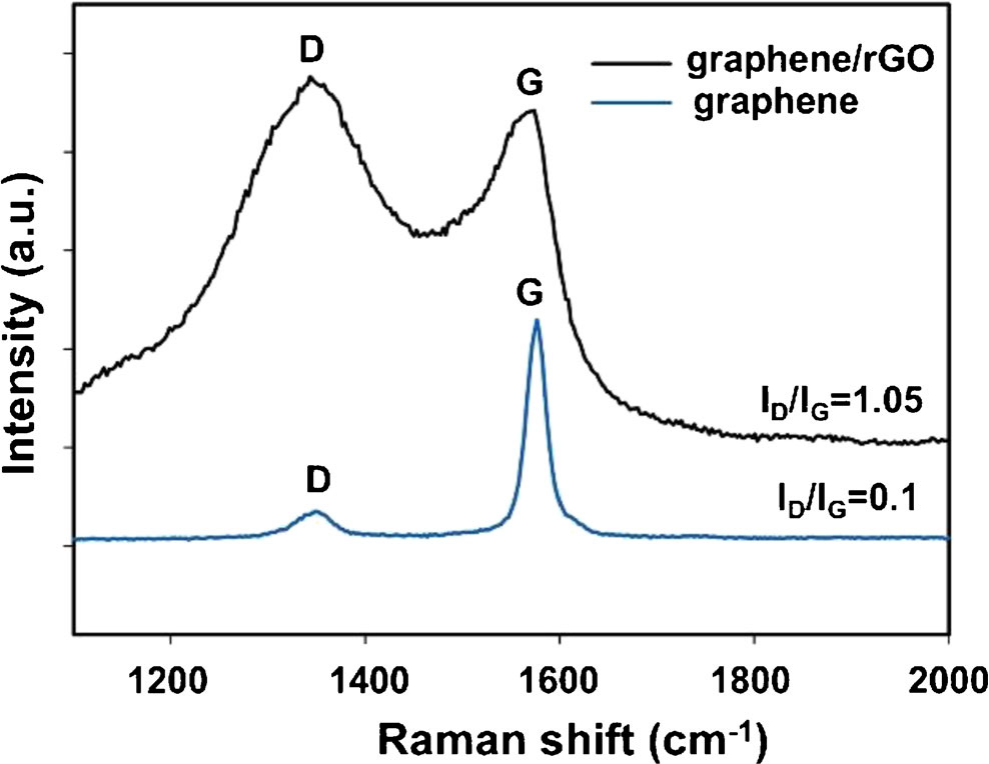
Raman spectra of graphene (blue) and graphene/rGO (black) dual-layer SPE

The Raman spectra of graphene and graphene/rGO SPE are shown in Fig. 2. In case of graphene (blue), there is a weak peak around 1346 cm^−1^ and a strong peak around 1574 cm^−1^. These can be attributed to the D and G band vibrations of graphene with intensity ratio of D to G band (I_D_/I_G_) equal to 0.1. Further, in case of graphene/rGO (black), wider peaks are observed around 1346 cm^−1^ and around 1574 cm^−1^. Here, intensity of D band is slightly higher than G band, resulting in a higher I_D_/I_G_ ratio of 1.05. The ratio I_D_/I_G_ indicates average distance between defective sites. It increases when the oxygen functionalities on GO are partly removed with electrochemical reduction [32].

**Fig. 3:**
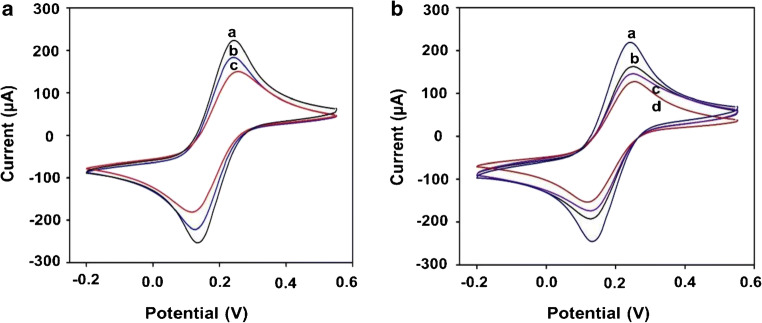
Cyclic voltammograms for the modification of SPE with **a** graphene/rGO (**a**), graphene (**b**) and graphene/GO (**c**); **b** graphene/rGO (**a**), antibody (**b**), BSA (**c**) and linker (**d**), and the CV was taken in 1 M PBS containing 10 mM [Fe(CN)_6_]^3−^ and 1 M KCl solution at a scan rate of 50 mV s^−1^

The CV of graphene and graphene/rGO dual-layer SPE were recorded from − 0.2 to + 0.5 V at a scan rate of 50 mV s^−1^ in 10 mM [Fe(CN)_6_]^3−/4−^ and 1 M KCl. The data was acquired at a working potential of 118 mV. Figure 3a shows a comparison in the voltammograms of graphene (blue), graphene/GO (red) and graphene/rGO dual-layer SPE (black). The modification of graphene with GO leads to a decrease in peak current. This is attributed to the long-range broken conjugated network of GO due to a large number of oxygen functional groups. Electrochemical reduction of GO leads to the formation of graphene/rGO dual-layer with higher peak currents compared with only graphene and graphene/GO-modified SPE. This is due to a combination of inherited electroactive sites from rGO and high conductivity of graphene [18].

For the fabrication of biosensor, voltammograms were recorded after each surface modification step. This includes graphene/rGO (blue), linker (red), antibody (black) and BSA (purple) as shown in Fig. 3b. The data was acquired at a working potential of 118 mV. It exhibit details relating to the kinetics of charge transfer of the redox probe [Fe(CN)_6_]^3−^ from solution to the electrode. This provides information about the interfacial properties of different layers on the surface. Assembly of linker decreases the anodic peak current (I_pa_) from 281.033 to 168.628 μA. This is due to an increase in the electron transfer resistance as it acquires available electroactive sites on rGO. The pyrene moiety binds to the rGO surface via non-covalent bonding (π-π interaction), whereas ester group forms an amide bond (covalent bonding) with the antibody [25]. The I_pa_ increases to 214.987 μA after the immobilization of antibody. This is attributed to the presence of free NH^3+^ groups (epsilon amines) present on the antibody. These groups accelerate electron transfer between electrode and [Fe(CN)_6_]^3−/4-^ system. Immobilization of BSA decreases the I_pa_ to 199.534 μA as it acquires free functional groups on the surface. This minimizes the chances of non-specific binding.

**Fig. 4:**
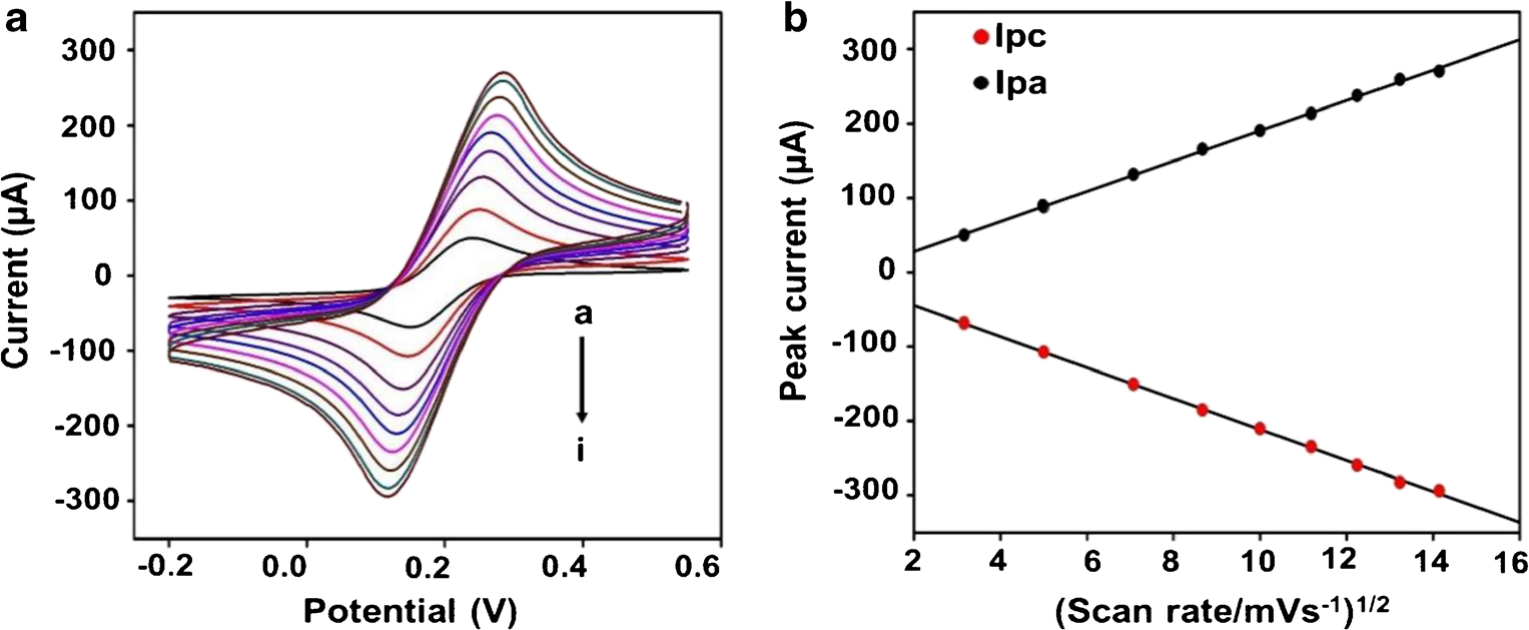
Scan rate studies of modified SPE **a** voltammograms under varying scan rates a-i (10, 25, 50, 75, 100, 125, 150, 175 and 200 mV s^−**1**^); **b** anodic (Ipa) and cathodic (Ipc) peak currents versus the square root of corresponding scan rate

Scan rate studies (10 to 200 mV s^−1^) of the modified SPE were performed to study redox process taking place at the surface. Peak to peak separation was found to be dependent on the scan rate indicating a quasi-reversible process. Both cathodic and anodic peak currents increase with an increment in scan rate (Fig. 4a). A linear correlation (*R*^2^ = 0.99) was obtained for current versus square root of scan rate (Fig. 4b). This is attributed to surface controlled diffusion of [Fe(CN)_6_]^3−/4-^ with no surface adsorption and is an important requirement for electrochemical biosensors [18]. Randles-Sevcik [25] equation was used for calculating the diffusion coefficient of the redox couple:

1 $$ {I}_{\mathrm{p}}=2\cdotp 69\times {10}^5A\ \sqrt{D}{\left(\sqrt{n}\right)}^3\sqrt{\upsilon }\ {C}_0 $$

where *I*_p_ is peak current of the electrode (in Ampere), *A* is the surface area in cm^2^ (0.126 cm^2^), *D* stands for the diffusion coefficient in cm^2^ s^−1^, *n* denotes the number of transferred electrons (*n* = 1), *υ* denotes the scan rate (V s^−1^) and *C*_0_ is the concentration of the redox couple (10 mM [Fe(CN)_6_]^3−/4−^). The value of D was found to be 1.414× 10^−6^ cm^2^ s^−1^ which is in close agreement with the value of 7.26 × 10^−6^ cm^2^ s^−1^in the literature [33].

The heterogeneous electron transfer rate constant (*K*_s_) was calculated for the modified SPE using the Lavrion model:

2 $$ \mathrm{Ks}=\frac{mnF\upsilon}{RT} $$

where *m* is peak to peak separation (0.12 V), *n* is number of transferred electrons (1), *F* is the Faraday constant (96,485.34 C mol^−1^), *υ* denotes scan rate (V s^−1^), *R* is universal gas constant (8.314 J mol^−1^ K^−1^) and *T* is absolute temperature (298 K). Value of K_s_ was calculated to be 0.23 s^−1^ at a scan rate of 50 mV s^−1^. This indicates fast electron transfer at the surface of SPE due to large electroactive sites and high conductivity of dual-layer.

### Optimization of sensor parameters

The following parameters were optimized: (a) incubation time of antibody and (b) concentration of linker; respective data and figures are given in the electronic supporting material. The following experimental conditions were found to give best results: (a) concentration of linker, 5 mM, and (b) incubation time of antibody, 16 h.

### Analytical performance of the biosensor

**Fig. 5:**
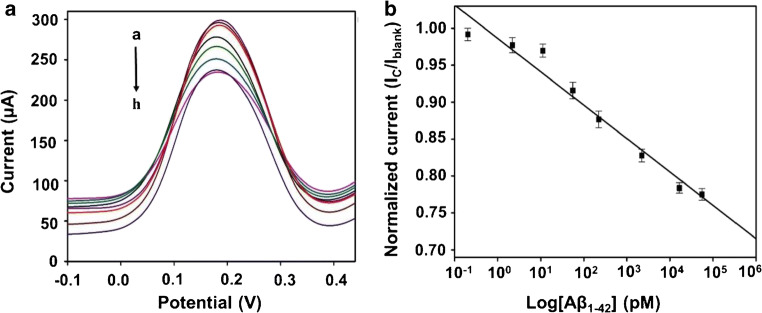
Analytical performance of the biosensor **a** DPV curves obtained for the biosensor for detection of different concentration of Aβ_1–42_ from a-h (0.2, 2, 11, 50, 220, 2200, 16,600 and 55,000 pM); **b** Calibration plot representing normalized current (I_C_/I_blank_) of DPV data as a function of Aβ_1–42_ concentration on a logarithmic scale (*n* = 3)

Under optimized condition, sensitivity of the biosensor was evaluated against a wide range of concentration from 0.2 pM to 55 nM using DPV. The results were acquired at a working potential of ~ 180 mV and a scan rate of 50 mV s^−1^. The current output of biosensor is shown as a function of different concentrations of Aβ_1–42_ in Fig. 5a. The peak current decreased with the increase in concentration. The calibration plot of normalized current (I_C_/I_blank_) versus concentrations (in pM) is shown in Fig. 5b with a linear regression coefficient (*R*^2^ = 0.97). The error bars were calculated based on 3 replicates of each experiment. The LOD was calculated as 2.398 pM using the following equation:

3 $$ \mathrm{LOD}=3.3\ast \left(\mathrm{SD}/\mathrm{SL}\right) $$

where SD is standard deviation of the normalized peak current value of lowest detectable concentration and SL is the slope of calibration plot. The excellent sensing response can be attributed to the sensitive structure of the biosensor. Graphene provides good electrocatalytic activity and electrochemical inertness [18], and rGO provides large electroactive sites. Pyr-NHS shows strong π-π interaction with the graphene/rGO dual-layer due to hydrophobic pyrenyl moiety base. Bioactive ester (NHS) group forms strong amide bond with the H31L21 antibody [25] resulting in a target-specific platform. However, fabrication process of the biosensor involves long incubation hours, particularly for antibody immobilization step. In addition, orientation of the antibodies on the surface is difficult to control and can reduce the capture efficiency [34].

### Specificity studies

**Fig. 6:**
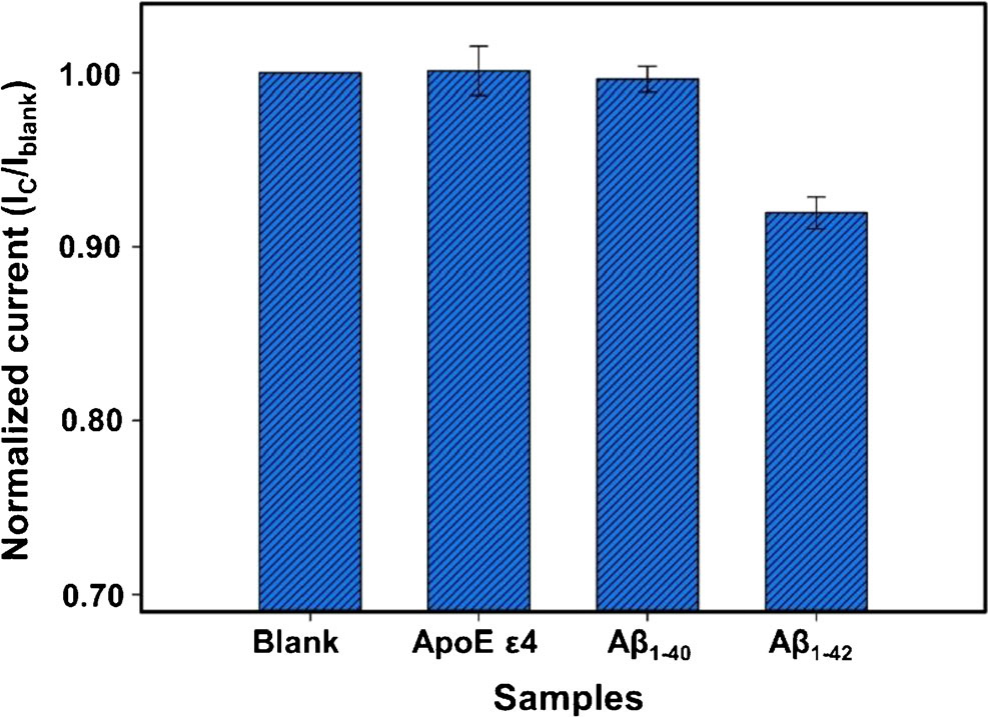
Specificity of the biosensor for the detection of 50 pM of Aβ_1–42_ with 500 nM of interfering agents: Aβ_1–40_ and ApoE ε4

The specificity towards Aβ_1–42_ was analysed using the DPV measurements. The modified SPE was incubated in blank (PBS buffer without protein), 50 pM of Aβ_1–42_ and 500 nM of Aβ_1–40_ and ApoE ε4 biomarkers. The higher concentration of interfering species was used to ensure the specificity of sensor in complex fluids such as plasma. The bar graphs obtained from the normalized peak currents were plotted as shown in Fig. 6. Only Aβ_1–42_ sample gave a significant decrease, whereas the interfering species were almost equivalent to the blank sample. These results illustrate high specificity of the biosensor towards the Aβ_1–42_ species.

### Plasma sample analysis

**Fig. 7:**
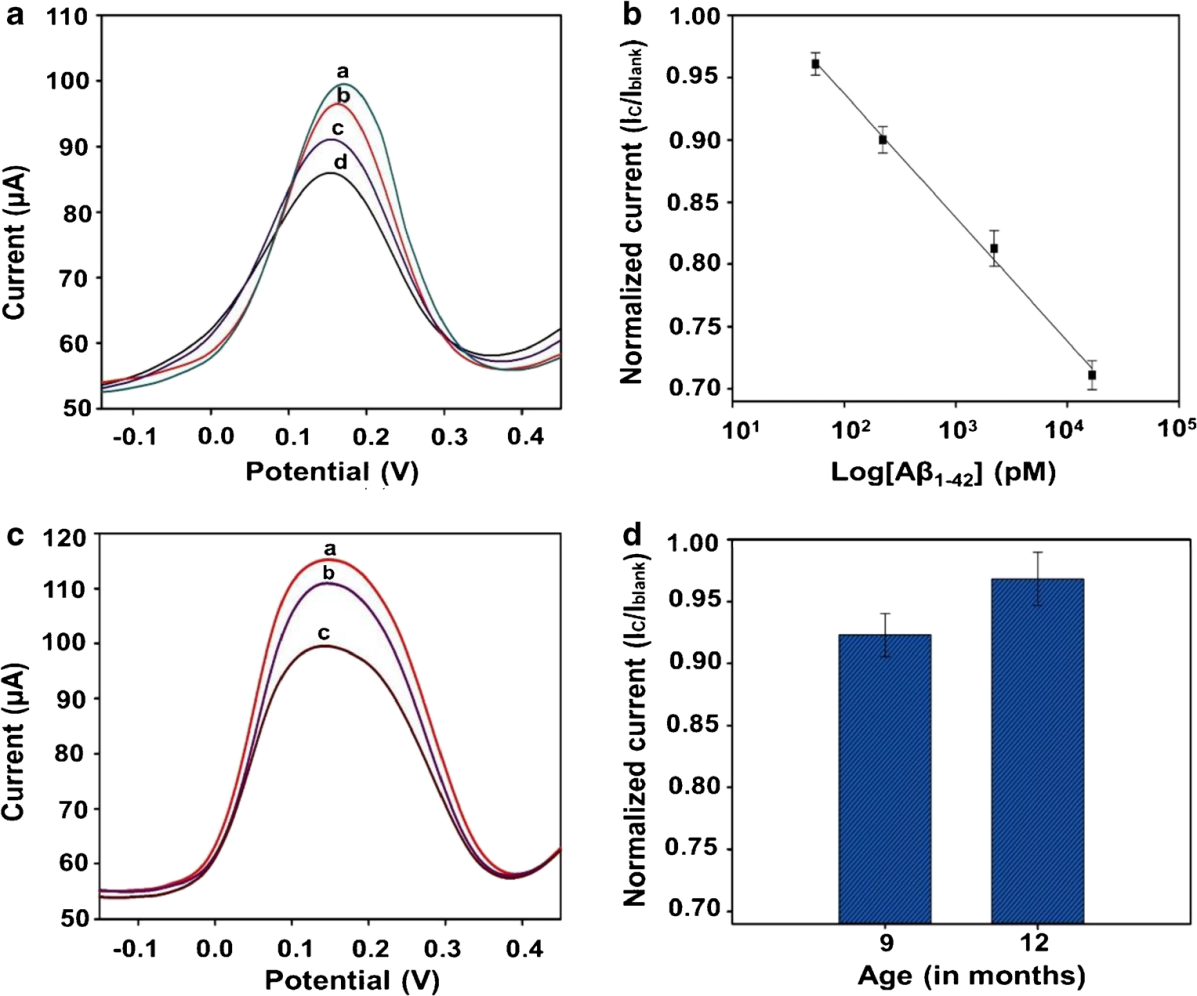
DPV responses from spiked concentration of Aβ_1–42_ (50 (**a**), 220 (**b**), 2200 (**c**) and 16,600 (**d**) pM) in human plasma (**a**); calibration plot of normalized current (I_C_/I_blank_) versus log of Aβ_1–42_ concentration (**b**); DPV responses for detection of WT (**b**) and Tg (**c**) mice compared with blank response (**a**); an age-based study with the two groups (9 and 12 months) of Tg animals (d) (*n* = 3)

Blood-based analysis of AD biomarkers is emerging as an alternative to the established strategies [35, 36]. This is attributed to the fact that blood sampling technique is less complex and minimally invasive and, therefore, can be applied to large communities. As a result, the biosensor was validated with blood plasma (in a series of two experiments) to check the applicability for bio-fluid analysis. In the first experiment, human plasma was spiked with known concentrations (50, 220, 2200 and 16,600 pM) of Aβ_1–42_. The DPV curves at varying concentration of spiked antigen and its calibration plot are shown in Fig. 7a, b. The sensing platform displayed high linearity in human plasma (*R*^2^ = 0.98).

In the second experiment, plasma samples obtained from 9 and 12-months-old WT and Tg mice were analysed without any pretreatment. The Tg mouse is an expression of a chimeric mouse and human amyloid precursor protein (Mo/HuAPP695swe). It also overexpresses a mutant human presenilin 1 (PS1-dE9) gene. Both mutations are associated with early-onset AD. Therefore, these animals are humanized models and produce human Aβ peptide species (e.g. Aβ_1–42_). These Aβ_1–42_ species can be detected by specific antibody that either recognize human or mice sequence or both of them [37]. For this reason, human Aβ_1–42_ antibody (H31L21) was used for validation of mice samples using the biosensor. The DPV results shown in Fig. 7c display a much larger shift in peak current for Tg mice as opposed to WT mice. This indicates a higher Aβ_1–42_ concentration in plasma of Tg mice. An age-based study was also performed as shown in Fig. 7d. As seen, a higher normalized current (*I*_C_/*I*_blank_) is observed in case of 12 months in contrast to 9-month-old mice plasma. This is attributed to the decrease in concentration of Aβ_1–42_ in the plasma of older mice with the progression of AD. However, a larger sample size (> 6) is needed for further validation of the biosensor before it can be employed for the determination of patients’ sample.

**Fig. 8:**
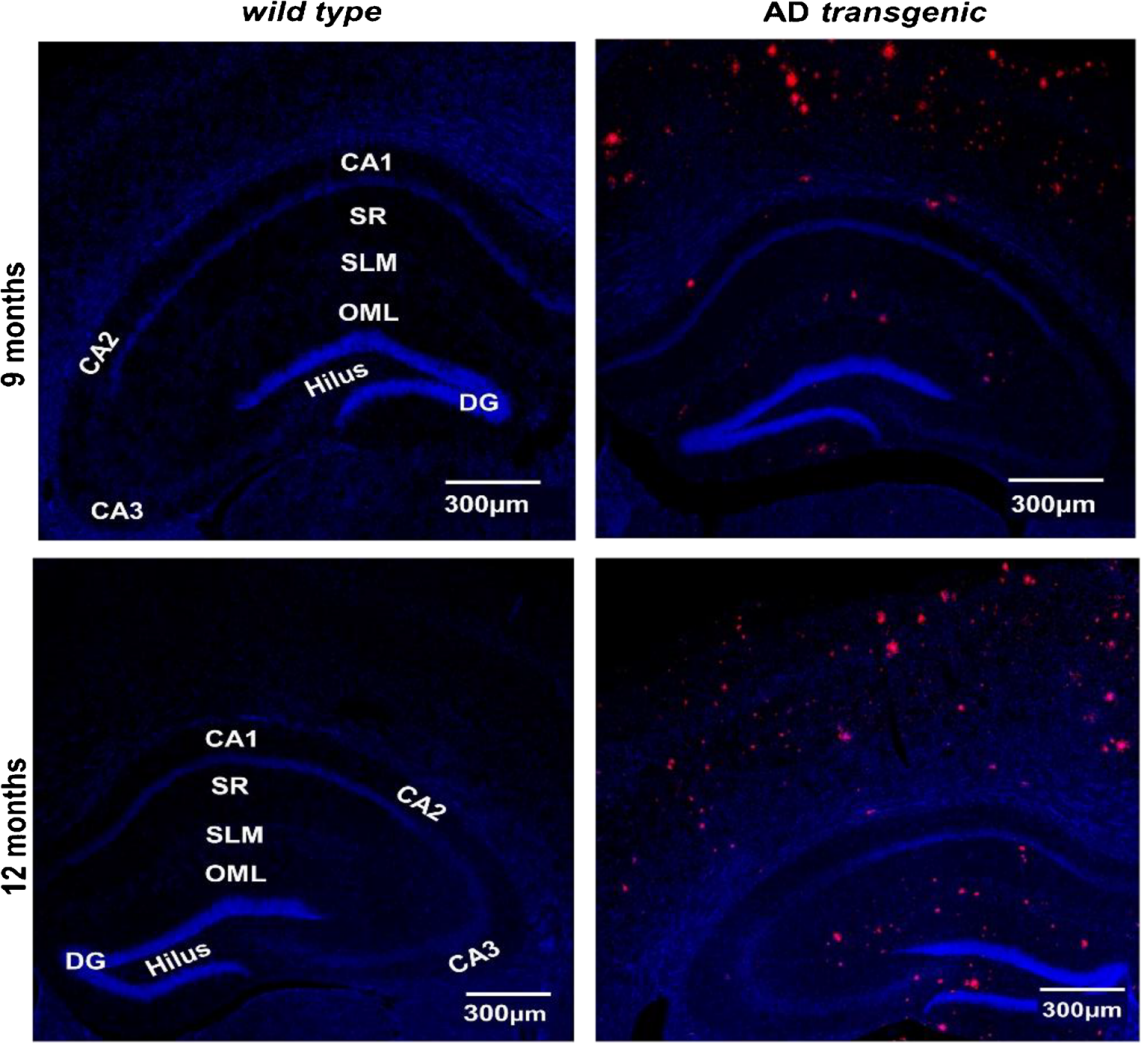
IHC data for the progression of AD pathology: An increase of human-specific Aβ_(1–42)_ (red) aggregation in cortex and hippocampal area; especially in stratum radiatum (SR), stratum lacunosum-moleculare (SLM) and outer portion of the molecular layer of dentate gyrus (OML) and the hilus of the dentate gyrus (DG), from 9 to12-months-old Tg compared with E littermates. Cornu ammonis (CA1, CA2, CA3 and hilus (CA4)) are subfield of the hippocampus; nuclei staining is in blue

The immunohistochemistry (IHC) data shows a higher accumulation of Aβ_1–42_ in both cortex and hippocampus region. It increases with age for Tg mice compared with WT mice (Fig. 8). This increase in Aβ plaques burden leads to decrease in plasma Aβ_1–42_ levels observed in Fig. 7 (d) [38]. The correlation in the IHC and sensing data further confirms the reliability of the platform for plasma sample analysis. The magnetic resonance imaging (MRI) data of the 12-month-old mice was collected. It also depicts Aβ plaques accumulation in cortex and hippocampus area of the brain (electronic supplementary information Fig. S5).

## Conclusion

In summary, graphene/rGO dual-layer SPE was developed for highly sensitive and label-free detection of Aβ_1–42_. The immobilization of antibodies was achieved via Pyr-NHS molecule. Its pyrene moiety binds to rGO via non-covalent bonding, and ester group forms strong amide bonds with antibody. The sensor depicted high specificity towards Aβ_1–42_ over interfering Aβ_1–40_ and ApoE ε4 species. It shows excellent performance for human and mice plasma analysis. Age-based study of mice samples exhibited a decrease in levels of Aβ_1–42_ with the disease progression (from 9 to 12 months old). This was attributed to the increased Aβ_1–42_ accumulation in 12-month-old mice shown by the IHC and MRI studies. However, the present study has few limitations. Firstly, the orientation of antibodies on the surface is random which can decrease the capture efficiency and affect the sensitivity of the platform. Secondly, fabrication process of the biosensor is time-consuming due to long incubation hours. Despite this, use of SPEs makes the fabrication process inexpensive and less complex with a possibility of mass production on a large scale. These can also be integrated with point of care devices to develop routine diagnostic tools for AD. For the future work, more comprehensive studies will be conducted using a larger sample size to verify the applicability of biosensor in identifying different stages of AD.

## Electronic supplementary material

ESM 1 (DOCX 923 kb).
