# Supplementary material for: A label-free biosensor based on graphene and reduced graphene oxide dual-layer for electrochemical determination of beta-amyloid biomarkers
Source: Mikrochim Acta. 2020 Apr 25;187(5):288. doi: 10.1007/s00604-020-04267-x (PMC7182627; doi:10.1007/s00604-020-04267-x)
Supplement: Supplementary file 1 — (DOCX 923 kb). [file 604_2020_4267_MOESM1_ESM.docx]

**Electronic Supporting Material**

**On the Microchimica Acta publication**

**entitled**

**A label-free biosensor based on graphene and reduced graphene oxide dual-layer for electrochemical determination of beta amyloid biomarkers**

**Jagriti Sethi ^a *^, Michiel Van Bulck ^b,c^, Ahmed Suhail ^a^, Mina Safarzadeh ^a^, Ana Perez-Castillo ^b,c^ and Genhua Pan ^a^**

**^a^ Wolfson Nanomagnetics Laboratory, School of Engineering, Computing and Mathematics , University of Plymouth, Devon, PL4 8AA UK**

**^b^Instituto de Investigaciones Biomédicas (CSIC-UAM), Arturo Duperier, 4. 28029 Madrid, Spain**

**^c^Centro de Investigación Biomédica en Red sobre Enfermedades Neurodegenerativas (CIBERNED), Valderrebollo, 5, 28031 Madrid, Spain**

**Characterization of the biosensor**

The SEM images display surface morphologies of the graphene and graphene/reduced graphene oxide (rGO) on carbon working electrode (Fig. S1). The images depict that modification of graphene with rGO did not have a significant effect on the surface confirming that no structural damage was done. This is in contrast to violent and deleterious chemical modification procedures used for graphene sensors which can damage its structure [1]. As a result, the unique electrical properties of graphene remain unaltered and a high redox current is observed for graphene/rGO dual-layers (Fig. 3).


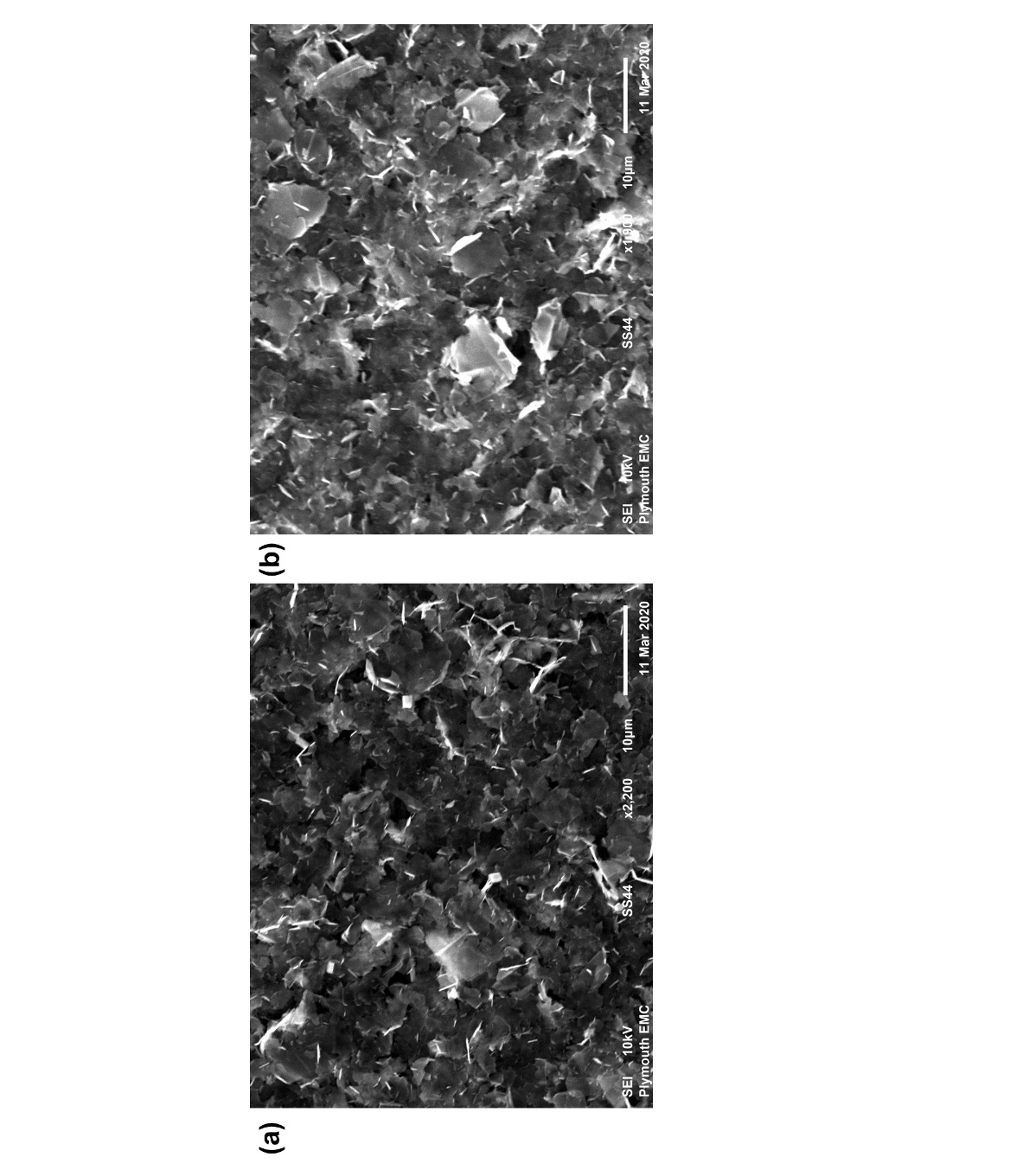


**Fig. S1** SEM images of (a) graphene and (b) graphene/rGO

X-ray photoelectron spectroscopy (XPS) was used to analyse the chemical states of the elements on graphene working electrode before (black) and after modification with rGO (green) (Fig. S2 (a,b,c)). The wide region spectra of graphene and graphene/rGO dual-layer surfaces show typical C (~284.5 eV) and O (~ 532 eV) peaks as shown in Fig. S2 (a). The atomic percentage (at%) of oxygen on the surface was calculated by XPS elemental analysis. It increases from 1.40 to 2.62 after modification with rGO. To further analyse the nature of C and O species, the C1s (Fig. S2 (b)) and O1s (Fig. S2(c)) regional high resolution spectra were plotted. The C1s of graphene demonstrate four peaks emerging from C-C/C=C in aromatic rings (~284.5 eV) , C-O (~286.14), C=O (~287.20 eV), and O-C=O (~291.3 6 eV) bonds [2]. After modification with rGO, an increase in C-O peak intensity is observed whereas no significant shift is seen in C=O and O-C=O peaks. This can be attributed to the reduced number of oxygen functionalities in rGO [3]. For further analysis, the O1s spectrum of the two surfaces were compared. The graphene/rGO surface can be deconvoluted into two peaks, namely C=O/ O-C=O (~531.5 eV) and C-O (~533.2 eV) [4] whereas graphene depicts a single lower intensity peak at ~533.2 eV. These results confirm the successful modification of graphene with rGO.


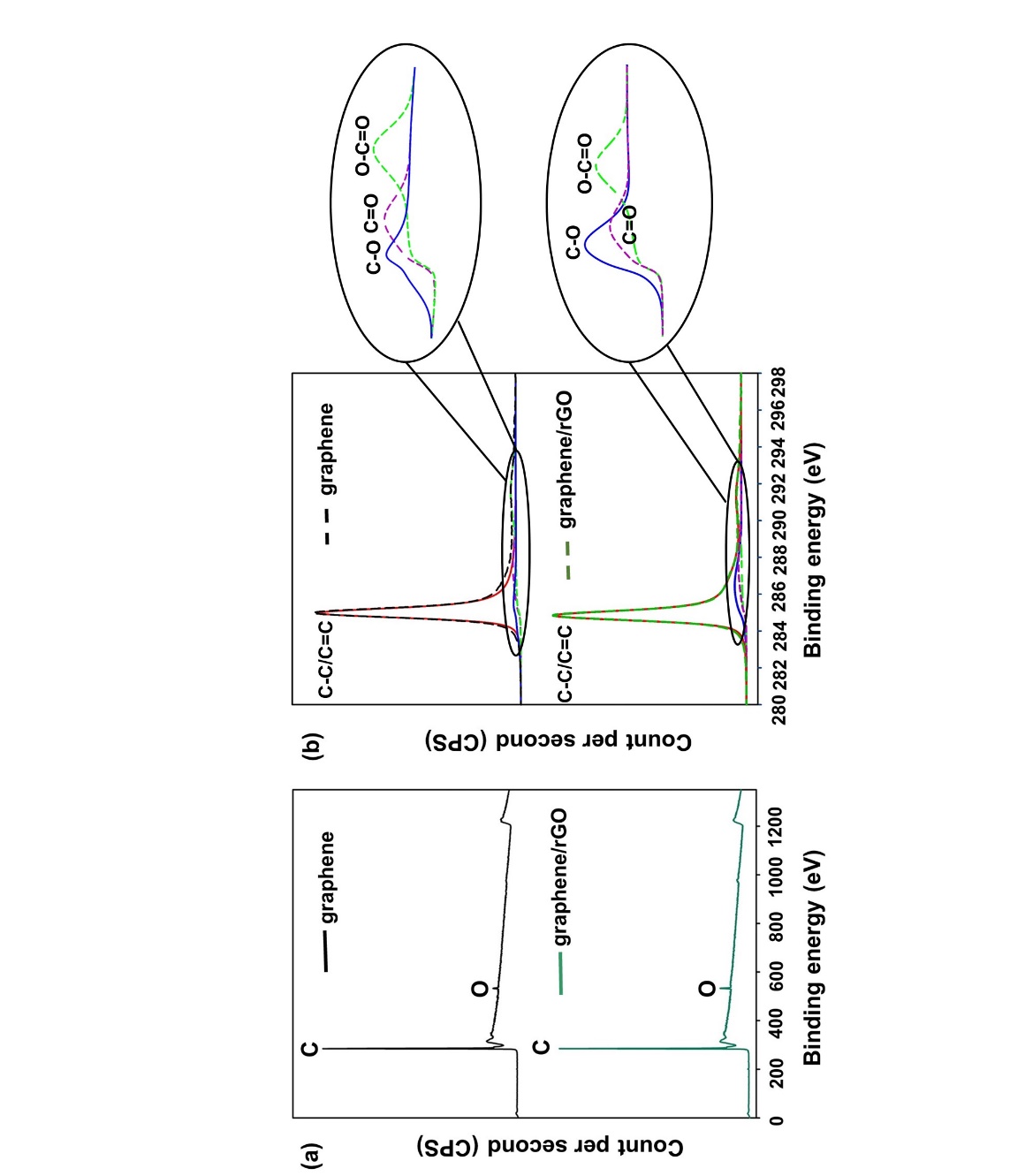


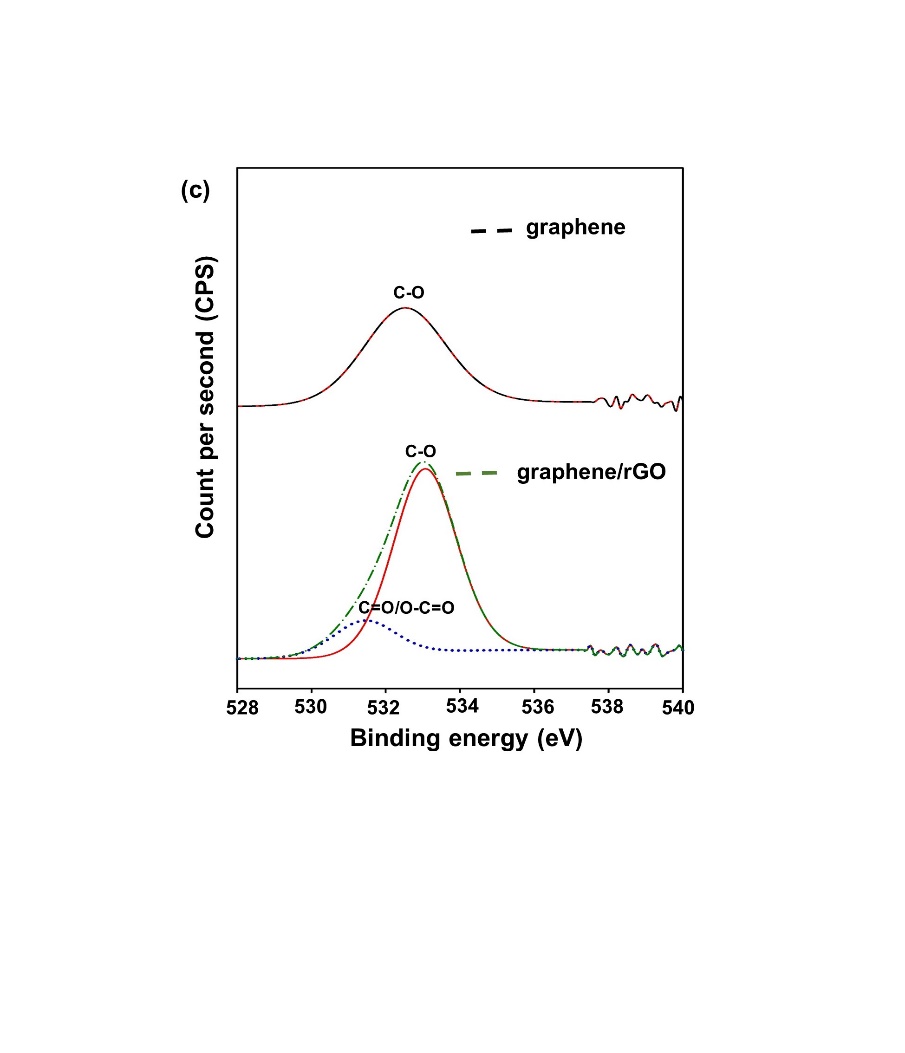


**Fig. S2** XPS spectrum for graphene (black) and graphene/rGO (green) showing (a) wide scan; (b) C 1s and (c) O 1s spectrum

**Optimization of sensor parameters**

Important experimental parameters such as incubation time of antibody and concentration of linker were optimised to obtain high sensitivity of the biosensor. Linker plays a crucial role on the effective immobilization of antibodies to enhance the sensitivity and lower the LOD [5]. Therefore, the effect of various linker concentrations (2, 3, 5, 7, 10 and 20 mM) on the normalized current for antibody (I_ab_/I_linker_) was studied (Fig S3 (a)). The results show that maximum value is obtained at 5 mM after which it steadily decreases with the increment in concentration. Therefore, concentrations higher than 5 mM decreases charge transfer of the redox probe. In order to further verify the above results, calibration plots for determination of Aβ_1-42_ (0.2 pM-55nM) using sensors modified with different linker concentrations were plotted (Fig. S4). The 5 mM linker concentration is found to be optimal with reproducible sensing response and is utilized in this work (Fig. 5). In addition, the effect of different incubation time of antibody (2, 4, 8, 16, 24 and 36 hrs at 4^o^C) on normalized current (I_ab_/I_linker_) is shown in Fig. S3 (b). As seen, the normalised current boosts drastically with the increment in time up to 16 hrs after which it starts decreasing. Consequently, 16 hrs is chosen as the optimal time for incubation of antibody.


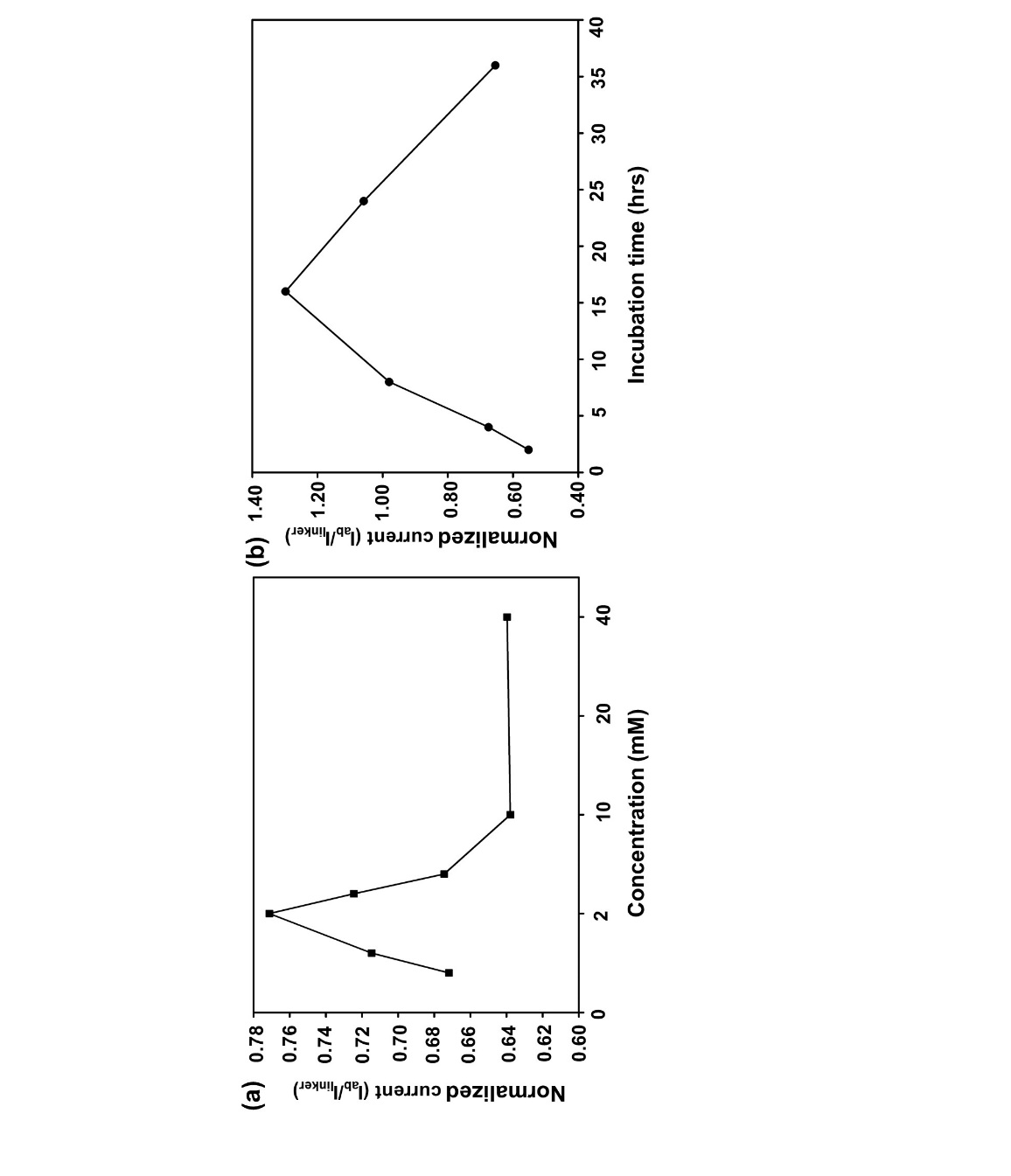


**Fig. S3** Effect of different linker concentration (a) and antibody incubation time (b) on the normalized current (I_ab_/I_linker_) response of the biosensor


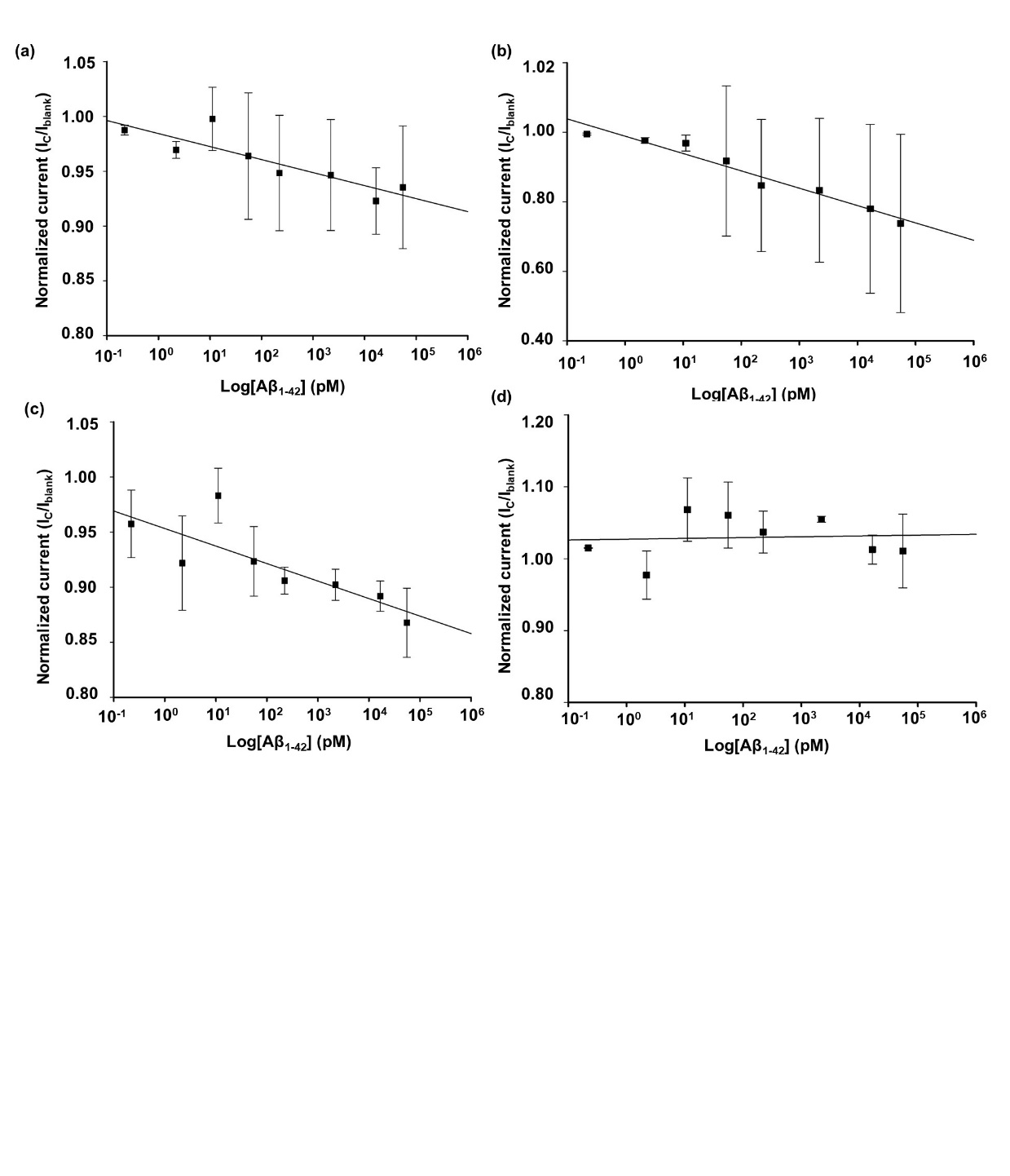


Fig. S4 Detection of various Aβ_1-42_ concentrations with biosensors modified with different linker concentrations namely, (a) 2mM; (b) 10mM; (c) 20mM; and (d) 40mM

**Magnetic Resonance Imaging (MRI)**

The MRI analysis was performed to see the β-amyloid load in the brain of 12 months old wt and tg mice (Fig. S5). Animals were anesthetized with a 2% isoflurane-oxygen mixture which was constantly regulated to maintain a breathing rate of 60 +/- 40 bpm. This was monitored with a respiration sensor placed under the abdomen of the animals. The mouse brain surface coil was mounted on the mouse cradle and placed over brain of the animal. Then, the animal was positioned in the magnet of the Bruker Pharmascan Biospect system. The acquisition of two sets of magnetization transfer contrast imaging were obtained. Finally, the MTR (magnetic transfer ratio) maps were calculated with a homemade software application written in Matlab (R2007a). The values were extracted from maps using the region of interest (ROI) with the Image J software.


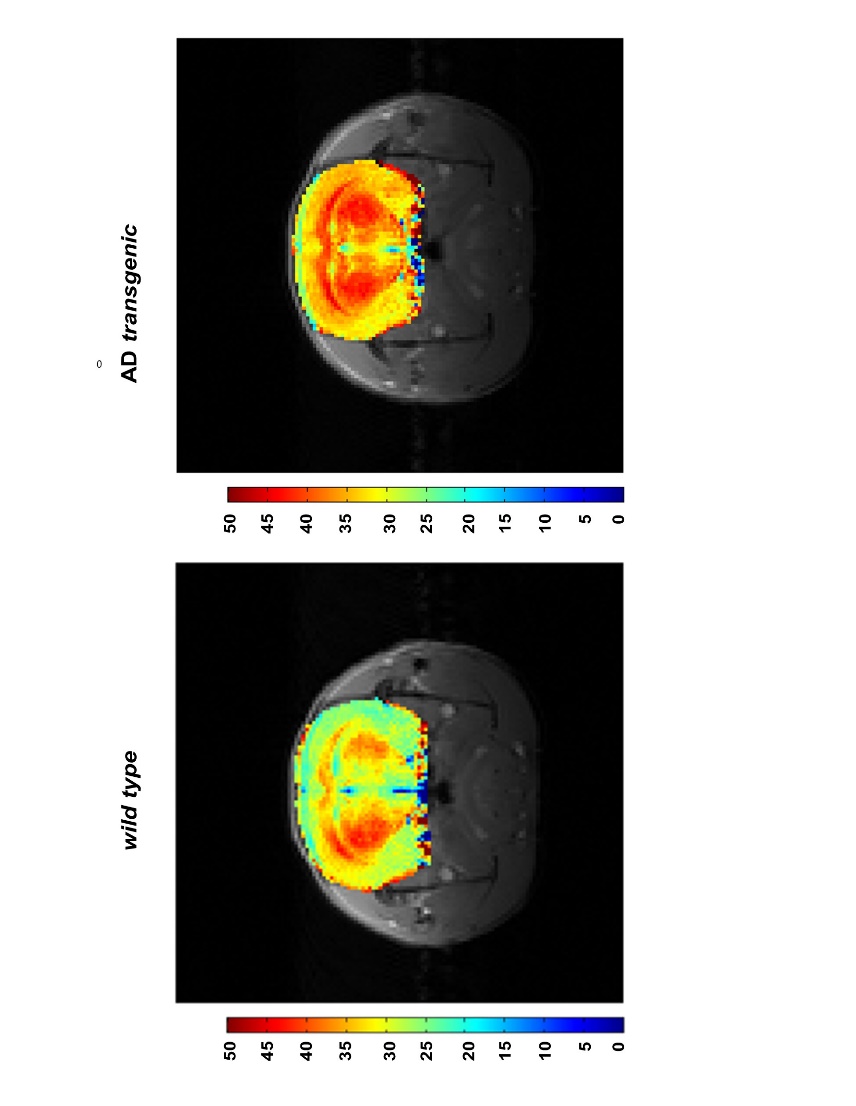


**Fig. S5** MRI depicting an increase of β-amyloid plaques accumulation in cortex and hippocampus of 12 months old tg (right) compared to wt (left). The ration of β-amyloid plaques accumulation is illustrated by a percentage value from 0-100% (from blue to red magnetic resonance spectra)

**References**

1. Teixeira S, Burwell G, Castaing A, Gonzalez D, Conlan S, Guy OJ Epitaxial graphene immunosensor for human chorionic gonadotropin. Sensors and Actuators B: Chemical (0). doi:<http://dx.doi.org/10.1016/j.snb.2013.09.019>

2. Xing Z, Ju Z, Zhao Y, Wan J, Zhu Y, Qiang Y, Qian Y (2016) One-pot hydrothermal synthesis of Nitrogen-doped graphene as high-performance anode materials for lithium ion batteries. Scientific Reports 6 (1):26146. doi:10.1038/srep26146

3. Li B, Pan G, Avent ND, Lowry RB, Madgett TE, Waines PL (2015) Graphene electrode modified with electrochemically reduced graphene oxide for label-free DNA detection. Biosensors and Bioelectronics 72:313-319. doi:<https://doi.org/10.1016/j.bios.2015.05.034>

4. Botas C, Álvarez P, Blanco C, Gutiérrez MD, Ares P, Zamani R, Arbiol J, Morante JR, Menéndez R (2012) Tailored graphene materials by chemical reduction of graphene oxides of different atomic structure. RSC Advances 2 (25):9643-9650. doi:10.1039/C2RA21447D

5. Balasubramanian K, Kern K (2014) 25th Anniversary Article: Label-Free Electrical Biodetection Using Carbon Nanostructures. Advanced Materials 26 (8):1154-1175. doi:doi:10.1002/adma.201304912
